# Rice Bran: From Waste to Nutritious Food Ingredients

**DOI:** 10.3390/nu15112503

**Published:** 2023-05-28

**Authors:** Bee Ling Tan, Mohd Esa Norhaizan, Lee Chin Chan

**Affiliations:** 1 Department of Healthcare Professional, Faculty of Health and Life Sciences, Management and Science University, University Drive, Off Persiaran Olahraga, Seksyen 13, 40100 Shah Alam, Selangor, Malaysia; 2 Department of Nutrition, Faculty of Medicine and Health Sciences, Universiti Putra Malaysia, 43400 Serdang, Selangor, Malaysia; 3 Natural Medicines and Products Research Laboratory (NaturMeds), Institute of Bioscience, Universiti Putra, Malaysia, 43400 Serdang, Selangor, Malaysia; 4 Biovalence Sdn. Bhd., 22, Jalan SS25/34, Taman Mayang, 47301 Petaling Jaya, Selangor, Malaysia; chanleechin@gmail.com

**Keywords:** cancer, diabetes, γ-oryzanol, *Oryza sativa*, rice bran, vitamin E

## Abstract

Rice (*Oryza sativa* L.) is a principal food for more than half of the world’s people. Rice is predominantly consumed as white rice, a refined grain that is produced during the rice milling process which removes the bran and germ and leaves the starchy endosperm. Rice bran is a by-product produced from the rice milling process, which contains many bioactive compounds, for instance, phenolic compounds, tocotrienols, tocopherols, and γ-oryzanol. These bioactive compounds are thought to protect against cancer, vascular disease, and type 2 diabetes. Extraction of rice bran oil also generates various by-products including rice bran wax, defatted rice bran, filtered cake, and rice acid oil, and some of them exert bioactive substances that could be utilized as functional food ingredients. However, rice bran is often utilized as animal feed or discarded as waste. Therefore, this review aimed to discuss the role of rice bran in metabolic ailments. The bioactive constituents and food product application of rice bran were also highlighted in this study. Collectively, a better understanding of the underlying molecular mechanism and the role of these bioactive compounds exerted in the rice bran would provide a useful approach for the food industry and prevent metabolic ailments.

## 1. Introduction

Rice is an important staple food consumed by more than half of the global population. It is cultivated in more than 100 countries, in which over 1 billion people depend on it for their livelihood, particularly in Asia. Based on the rice production quantity data (2020) estimated by the Food and Agriculture Organization of the United Nations (FAO), the world production of paddy rice in 2020 was about 758 million tons. The demand for rice is projected to remain strong in the next few decades due to the increasing population [1]. Thus, the rice industry will remain sustainable for a long time, and rice by-product production will remain high [2].

Rice is primarily consumed as white rice, a refined grain that is generated from the rice milling process which removes the germ and bran and leaves the starchy endosperm. Rice bran is a vital by-product generated by the rice milling industry, which is obtained while polishing and milling brown rice into white rice (polished rice) [3]. Rice bran is comprised of 12–18% oil, which has beneficial nutraceuticals, for instance, phospholipids, oryzanol, squalene, phytosterols, tocotrienols, and tocopherols [4].

Emerging evidence suggests that rice bran and rice bran by-products may exert beneficial health effects against metabolic ailments and oxidative stress [5]. This favorable effect has been associated with the bioactive components that are present in the rice bran. A recent finding found that γ-oryzanol-enriched rice bran oil was shown to decrease cholesterol and low-density lipoprotein cholesterol (LDL-C) levels in hypercholesterolemic patients, suggesting that γ-oryzanol-enriched rice bran oil may decrease cardiovascular disease (CVD) risk factor [6]. In addition, vitamin E and some phenolic compounds in rice bran are also shown a potentially beneficial effect, for instance, anticancer and antioxidative activity [7]. Nevertheless, rice bran is often used as animal feed or discarded as waste [8]. The disposal of rice bran has caused an adverse outcome. Research evidence has revealed that unused rice by-products are usually burnt in the field and hence may cause environmental problems, for instance, smog formation, air pollution, and economic waste [9]. Due to the increase in the availability and acceptability of the by-products from the food processing industries, it has now been considered the predominant source of functional ingredients in secondary food processing industries [10]. Hence, this review aimed to highlight the role of rice bran in metabolic ailments. The bioactive constituents and food product application of rice bran were also discussed in this study.

## 2. Rice Demands

**Figure 1:**
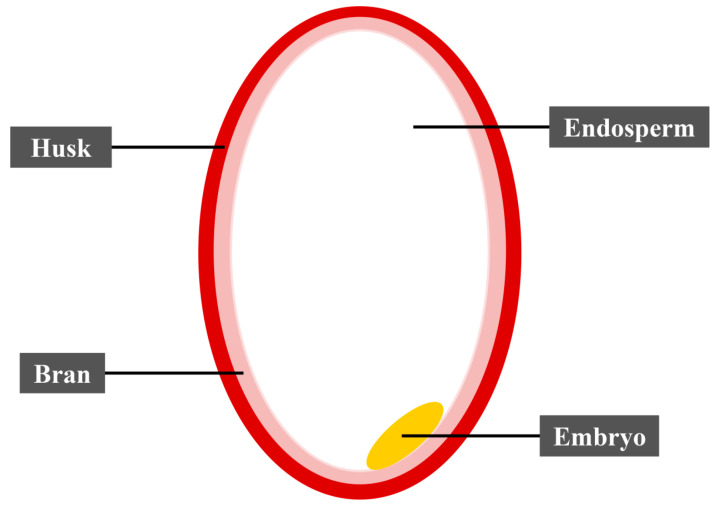
Structure of rice grain.

Maize, rice, and wheat are widely grown crops worldwide. Of these major crops, rice is eaten mainly as milled whole grain. It is often marketed in three grain sizes, namely short or round grain (less than 5 mm long), medium grain (5 to 6 mm long), and long grain (over 6 mm long) [11]. Rice (*Oryza sativa* L.) is widely grown rice and provides more than 50% of the dietary caloric supply and substantial protein intake for nearly 520 million people living in poverty in Asia. Rice intake in urban dwellers is steadily increased in Sub-Saharan Africa, with per capita consumption doubled since 1970 [12]. Other countries such as Latin America and the Caribbean regions are also shown steady growth in rice consumption [12]. Therefore, rice is crucial for the nutrition of large parts of the populations in the Caribbean, some regions in Latin America, the Asia Pacific region, and Africa. Importantly, it is also the main source of employment and income for over 200 million households across countries in developing countries [13]. Figure 1 shows the structure of rice grains.

Nearly 90% of the rice is produced and consumed in Asian Regions such as India, Bangladesh, Japan, China, Indonesia, and Vietnam, which contribute about 80% of the global production and consumption [14]. Increased rice demands depend on various factors such as (1) growth in population; (2) the change in prices relative to substitute crops; and (3) the level of per capita income [15]. The global population is expected to reach 11.2 billion in 2100, 9.8 billion in 2050, and 8.6 billion in 2030. Because 83 million people are increasing annually, the upward trend in population size is projected to continue. China and India remain the two most populous countries, contributing 19 and 18% of the total world population, respectively [16]. Table 1 shows the population of rice-producing and consuming countries in Asia between 1995 to 2025. In this regard, rice production is projected to increase until 2050. Similarly, a study by Lim et al. [17] revealed a steady increase in rice demands in the coming years, hence the rice industry will remain strong in the next few decades. It has been suggested that rice by-products will substantially increase by 2050. Therefore, the applications of rice by-products have remained crucial.

## 3. Production of Rice Bran

Rice bran is a by-product obtained from milling, contributing to nearly 10% of whole grain weight. It is comprised of the nucellus, seed coat, subaleurone, aleurone, pericarp, germ, and a minor fraction of endosperm [19]. The rice bran production is projected to increase annually [2]. In 2014–2015, the rice bran production is nearly 23.80 million tons worldwide [20].

During the rice milling process, the rough rice or paddy is initially cleaned to separate particulates and contaminants such as stones, straw, weed seeds, and soils. The husk is removed from the rice paddy by friction in shellers. The brown rice and husks are separated by aspiration and the paddy remaining with the brown rice is separated by a paddy separator to further separate the broken rice from unhulled [21]. The brown rice goes to a milling machine, known as huller, in which the bran layer is removed from the germ and leaves a nearly white kernel. The amount of bran removed is usually 8–10% of the total paddy weight and varied according to the degree of milling, type of milling system, pretreatment before milling, and rice variety [22].

## 4. Effect of Processing on the Rice Bran

### 4.1. Lipid Oxidation in Rice Bran

Rice bran has a shorter shelf-life than refined white rice due to the production of free fatty acids during storage [23]. Proper storage and packaging can extend the shelf-life of white rice by up to a decade, compared to only about 1 year in the rice bran fraction [24]. Indeed, rice bran can store longer by deactivating the lipase enzymes that are responsible for the denaturation of anti-nutritional factors and hydrolytic degradation of bran components [25]. Rancidity caused by lipase limits the utilization of rice bran as a food source. These enzymes are activated immediately after the milling process [26]. The oil is hydrolyzed into glycerol and free fatty acid when the oil is not extracted after milling [27]. Subsequently, free fatty acids change the functional properties of bran, produce a soapy taste and off-flavor, decrease pH, and trigger bran acidity [20]. In grains, the enzymes are found in the testa-cross layer of the grains, while the oil vacuoles are present in the sub-aleurone, germ, and aleurone layers [20]. After the milling process, the oil is exposed to lipases and thus breakdown into free fatty acids at the rate of 5–7% of the oil weight per day [28]. The previous study has revealed that more than 70% of the free fatty acids were found in a single month of bran [29]. Rice bran oil has 2–4% of free fatty acids at the time of milling. The rancid oil and its related products are toxic and thus are regarded as an anti-nutritional factor that decreases the shelf-life of the bran [30]. For instance, oil with more than 10% free fatty acids is unfit for human consumption. The oil with less than 5% free fatty acid is desirable for rice bran oil production due to the increased free fatty acids that may result in high refining losses [31].

**Figure 2:**
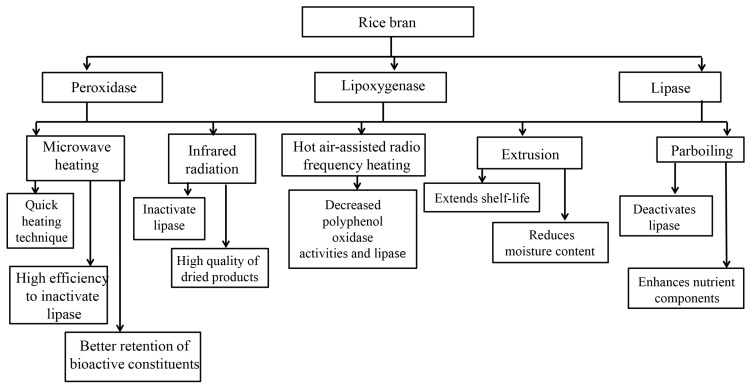
Stabilization techniques and their effects on the rice bran.

The activities of lipases are highly dependent on water activity, time, pH, temperature, and moisture [32]. A study has shown that activity of the enzyme was active up to 40 °C and markedly reduced to 65% at 60 °C followed by gradually decreasing [33]. In addition to lipases, microbial lipases also destroyed the nutritional quality of the oil. The hydrolytic rancidity severely influences the palatability and nutritive components of rice bran. Trypsin inhibitor is another endogenous enzyme, which can form a stable complex with proteolytic pancreatic enzymes [34]. Phytate accounts for 85% of the total phosphorus content of cereal grains [35]. The digestibility and bioavailability of nutrients are reduced by forming complexes with digestive enzymes, protein, minerals, and amino acids such as histidine, arginine, methionine, and lysine. Hydrolytic rancidity limits the application of rice bran in foods as well as decreases the quality [36]. Hence, the rice bran stabilization has drawn the attention of the producers. Indeed, the stabilization of rice bran is a primary step to developing its value-added utilization by preventing undesirable outcomes such as deterioration of nutrients, refining loss, off-flavor, and rancidity [37]. After proper stabilization, rice bran could be served as a good source of proteins, essential fatty acids, ferulic acid derivatives, and tocopherols [20]. Figure 2 shows the stabilization techniques and their effects on the rice bran.

### 4.2. Stabilization Techniques of Rice Bran

Several stabilization techniques such as chemical methods, enzymatic treatment, refrigeration or other physical methods, hydrothermal or parboiling treatment, ohmic heating, infrared radiation, microwave heating, extrusion cooking, and moist and dry heating have been employed immediately upon milling process to prevent rancidity in rice bran. An optimized stabilization procedure is critical for the food industry to minimize the loss of bioactive components [25]. Infrared radiation and microwave heating are regarded as alternative energy sources with high heat efficiency in a short processing time without affecting the quality of rice bran [38]. Heat stabilization is performed commercially by dry or wet heating procedures, namely microwave, dry extrusion, drum drying, and hot air [39].

Microwave stabilization is a quick heating technique with high efficiency to inactivate lipase and had better retention of bioactive constituents [40,41]. Likewise, infrared radiation is also an alternative processing procedure to achieve efficient drying efficiency as well as inactivate lipase in rice bran without influencing the quality [38]. In a study focused on stabilization treatments, Yu et al. [42] compared eleven rice bran stabilization procedures including five non-heating treatments and six heating treatments by evaluating the lipase activities. The data showed that ultraviolet irradiation, extrusion, and microwave heating are suitable for industrial processing. Yu et al. [42] further demonstrated that ultraviolet irradiation is a potential, energy-saving, and convenient stabilization procedure without affecting the nutrient components and oil quality. Infrared-radiation stabilization has become a crucial procedure in the food industry in recent decades due to its numerous advantages such as the clean operational environment, a uniform temperature, easy control of the process parameters, high quality of dried products, low drying time, low energy cost, and low capital cost [43]. The previous finding suggests that infrared radiation heating suppressed the deterioration of rice bran during short-term storage [44]. The study demonstrated that after 20 days of storage, the enzyme activities such as peroxidase, lipoxygenase, and lipase are all decreased in freshly milled paddy rice bran treated with infrared radiation [44]. Infrared radiation can decrease the hydroperoxides and oxidation of rice bran, which can control the increase in peroxide value until the 15 days of storage [44]. The study found that palmitic acid, oleic acid, and linoleic acid were well maintained after infrared radiation heating throughout the whole storage period [44]. Intriguingly, the aroma of fresh rice bran was acceptably preserved with an abundant amount of alkanes and aldehydes [44]. This finding suggests that infrared radiation processing can effectively stabilize the rice bran in terms of aroma, enzyme activities, and fatty acid profiles [44]. Irakli et al. [25] compared microwave heating, dry heating, and infrared radiation on the functional, anti-nutritional, and nutritional content of rice bran. The study showed that rice bran stabilized with microwave heating, infrared radiation, and dry heating can reduce trypsin inhibitors, saponins, oxalate, and phytates effectively [25]. However, total phenolic content and vitamin E in rice bran were significantly reduced by microwave heating, infrared radiation, and dry heating. Infrared radiation enhanced the water solubility index and swelling power of rice bran, whereas microwave heating improved the foaming properties [25]. Infrared radiation heating was shown to be the most effective in prolonging the shelf-life and inactivating lipase activity of rice bran compared to microwave heating and dry heating, suggesting that rice bran treated with infrared radiation could stabilize rice bran by improving some functional properties and inactivating lipase [25]. Hot air-assisted radio frequency heating is another stabilization technique for rice bran [45]. Hot air-assisted radio frequency heating (high temperature = 110–115 °C for 6 min and low temperature = 100–105 °C for 15 min) decreased polyphenol oxidase activities and lipase of fresh rice bran [45]. The previous finding demonstrated that hot air-assisted radio frequency stabilized rice bran showed higher antioxidant activity and free flavonoid content, and more intact microstructure than extruded rice bran [45]. Extrusion cooking is a process of heating the food under high pressure that caused a reduction of moisture content in dried and cooked food and thus extends the shelf-life of rice bran [46]. A recent study found that the combination of infrared heat and vacuum decreased the fatty acid hydrolysis while preserving the bran’s essential fatty acids and bioactive compounds including tocols [36]. The study further demonstrated that the stabilization of rice bran improved the shelf life (130 days) compared to only 35 days in fresh rice bran [36]. These observations imply that bioactive compounds, for instance, oryzanol and tocols in the stabilized rice bran were well preserved with nearly four times longer shelf life compared to fresh rice bran [36]. Moreover, parboiling deactivates the lipase and thus causes the leaking of water-soluble vitamins and minerals to the endosperm from the bran, which enhances the nutrient components [47]. Nonetheless, this process destroyed the antioxidants and changed the nutrient profiles that slightly differ from the heat-stabilized rice bran [48].

## 5. The Role of Rice Bran in Health and Chronic Diseases

**Figure 3:**
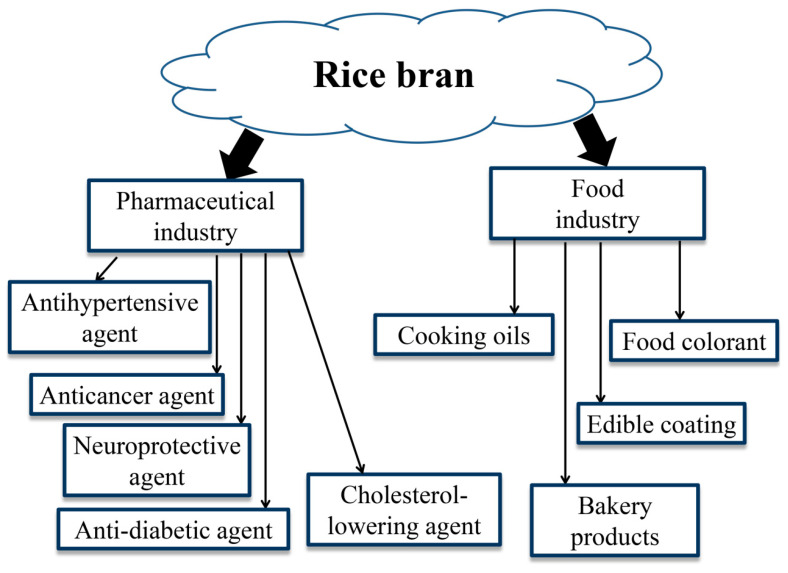
The applications of rice bran in pharmaceutical and food industries.

Empirical evidence suggests that rice bran exerts antioxidant activity and numerous bioactive components, for instance, phytosterols, γ-oryzanol, γ-aminobutyric acid (GABA), dietary fiber, and vitamin E, which are beneficial to human health (Table 2). These bioactive constituents were found to have antioxidative activity and health-beneficial properties including anti-inflammatory, anti-cancer, hypotensive, anti-diabetic, cardioprotective, and hypocholesterolemic effects (Figure 3). In addition, pigmented rice varieties have also received attention from consumers due to their bioactive constituents. It has been revealed that these bioactive constituents are predominantly present in the outer layer of rice grains. Several rice cultivars contain pigments in their pericarp and seed coat which exhibit colors of the pigments on the outer layer. Proanthocyanidins and anthocyanins are thought to be the predominant functional components found in colored rice bran, which have health-beneficial properties and antioxidant activity.

### 5.1. Hypertension

Hypertension is one of the risk factors for cardiovascular disease (CVD), affecting 1.13 billion people worldwide [63]. Hypertension is also known as raised or high blood pressure, in which the blood vessels have persistently raised pressure [63]. In particular, hypertension is treated with angiotensin-converting enzyme (ACE) inhibitors [64]. Angiotensin I-converting enzyme is a crucial enzyme in the mediation of blood pressure via two different reactions in the kinin nitric oxide system (KNOS) and renin–angiotensin–aldosterone system (RAAS) [65]. ACE plays a crucial role in the modulation of blood pressure. Angiotensin-I is converted to angiotensin-II by modulating ACE and subsequently leading to elevation of blood pressure and arterial constriction. While numerous efforts have been made in recent decades to enhance the available therapeutic approach, this therapy seems to be not effective due to adverse outcomes. For instance, many synthetic ACE inhibitors including lisinopril, ramipril, fosinopril, enalapril, and captopril cause unwanted adverse outcomes including skin rash, taste disturbance, and coughing [66]. Therefore, the discovery of new ACE inhibitors from natural resources has drawn interest among scientists from industry and academia [67].

Rice bran has beneficial biochemical and antioxidant effects on many diseases including dementia, cardiovascular disease (CVD), Alzheimer’s disease, cancer, and diabetes [68]. Rice bran was found to improve hypertension in animals based on numerous studies. Feeding stroke-prone spontaneously hypertensive rats with fermented rice bran (40 mg/kg body weight) improved high blood pressure after 6 h of administration [69]. In addition to its effects on blood pressure, the study demonstrated the roles of fermented rice bran in modulating glucose metabolism [69], demonstrating the enormous functional potential of fermented rice bran. In the double-blind, randomized, placebo-controlled study, a significant reduction in systolic blood pressure was found at 12 weeks in individuals with grade 1 hypertension or high–normal blood pressure supplemented with processed rice bran compared to the placebo group [70]. This observation suggests that daily intake of rice bran supplement containing processed rice bran containing 43 μg Leu-Arg-Ala/day could improve mildly increased blood pressure [70]. Table 3 summarizes the effects of rice bran and its derived compounds on metabolic ailments.

Rice bran bioactive peptides constitute alternatives for this, which may serve directly as ACE inhibitors [90]. Protein-rich food is digested by proteases and fermented by microorganisms, for instance, *Lactobacillales*, to produce anti-hypertensive peptides [91]. Rice bran protein not only possessed antioxidant properties, but they have also facilitated the mediation of blood pressure. An emerging role of peptides derived from rice bran protein in response to hypertension has been demonstrated [90]. Emerging research evidence indicates that rice bran protein hydrolysate (<3 kDa) exerts angiotensin-converting enzyme (ACE) inhibitory activity. A study by Piotrowicz et al. [71] evaluated the ACE inhibitory activity and blood pressure-lowering effect of rice bran protein hydrolysates *in vivo*. The study showed that fractions <3 kDa obtained from alcalase demonstrated a potent ACE inhibitory activity such as rice bran protein hydrolysate 1A < 3 kDa and 2A < 3 kDa [71]. Oral administration of 80 mg/kg rice bran protein hydrolysates 1A < 3 kDa significantly decreased the systolic blood pressure (SBP) in spontaneously hypertensive rats, in which the maximum reduction was reached at 8 h after administration [71]. These data suggest that rice bran protein hydrolysates produced from the food industry after treatment with alcalase may serve as a source of bioactive peptides, with potential action on hypertension [71]. A study by Shobako et al. [72] found an anti-hypertensive peptide, Leu-Arg-Ala, from thermolysin-digested rice bran. The study showed that Leu-Arg-Ala exerts strong vasodilating and anti-hypertensive effects [92]. Feeding spontaneously hypertensive rats (SHR) with Leu-Arg-Ala (0.25 mg/kg) decreased SBP levels, suggesting that Leu-Arg-Ala is a potent anti-hypertensive peptide derived from rice protein [72]. An animal study has also shown that Leu-Arg-Ala exerts vasorelaxant activity in the mesenteric artery isolated from SHR [92]. Intriguingly, the suppression of ACE is often linked to antihypertensive activity as the inhibitors, which can lower blood pressure and modulate the constricted blood vessels [93]. In this regard, rice bran protein hydrolysates and rice bran protein hold great promise and may serve as an ingredient for the treatment and prevention of hypertension.

### 5.2. Cancer

Cancer has become the second leading cause of death globally, accounting for approximately 9.6 million deaths, or 1 in 6 deaths in 2018 [94]. In particular, nearly 30–50% of cancers could be prevented [94]. Oxidative stress is hypothesized to be linked to cancer. During the initial stage of carcinogenesis, increased reactive oxygen species (ROS) promote susceptibility to mutagenic agents and mutation rates and subsequently cause DNA damage [95]. Furthermore, the increase in ROS was also found in tumor growth through modulation of ligand-independent transactivation of receptor tyrosine kinase [96], which can lead to invasion and metastasis of cancer cells. Oxidative stress induces various transcriptional factors including NF-κB, nuclear factor E2-related factor 2 (*Nrf2*), and Wnt/β-catenin, and subsequently stimulates inflammatory pathways [97].

Many studies have highlighted the beneficial role of rice bran on cancer in in vivo and in vitro [5]. Rice bran suppresses the growth of cancerous cells via a few mechanisms. In cancer cells, rice bran scavenges free radicals, regulates the antioxidant pathway, induces apoptosis, and modulates Wnt/β-catenin and inflammation signaling pathways [5]. Evidence from in vitro study has shown that cyanidin 3-glucoside derived from rice bran reduced the percentage of cell survival and decreased the expression of p-FAK and β-catenin in prostate cancer PC-3 cells (Table 3) [73]. Cyanidin 3-glucoside is mainly presented as a natural aglycone form of anthocyanins [73]. The study further revealed that cyanidin 3-glucoside derived from rice bran suppressed β-catenin and its signaling cascade, thereby leading to the suppression of dissociation and translocation from the cell periphery, which plays a crucial role in the mediation of cell differentiation signaling via epithelial–mesenchymal transition (EMT) process [73]. These data suggest that cyanidin 3-glucoside derived from rice bran reduces the distribution at the cell periphery and decreased p-FAK levels in the PC-3 cells, which may decrease cell–matrix interaction [73].

Rice bran polysaccharides have drawn interest from the pharmaceutical industry. Previous studies have demonstrated that rice bran polysaccharides suppressed several cancers including Lewis lung carcinomas [98], mammary tumors [99], and gastrointestinal cancer [100]. Han et al. [74] compared different groups of H1299 non-small-cell lung cancer-bearing mice that were treated with polysaccharides from the fermentation products of *Ganoderma sinense*–defatted rice bran, rice bran polysaccharides, and full-fat rice bran, respectively. The data demonstrated that *Ganoderma sinense* defatted rice bran and *Ganoderma sinense* rice bran polysaccharides showed a better inhibitory activity on H1299 non-small-cell lung cancer compared to *Ganoderma sinense* full-fat rice bran [74].

Rice bran arabinoxylan compound is a water-soluble modified arabinoxylan with xylose formed from the main chain and an arabinose polymer conjugated to a side chain [101]. MGN-3/BioBran is a bio-modified rice bran polysaccharide fermented using the carbohydrate hydrolyzing enzymes from shiitake mushrooms [102], which has been shown as a promising natural adjuvant to existing immunotherapy for cancer [103] and suppressed colorectal cancer, myeloma, and leukemic cells [104] via antioxidant properties [105]. The preclinical studies indicate that the rice bran arabinoxylan compound can mediate several cytokines production, for instance, TNF-α, IL-17, IL-10, IL-6, and IL-1β [106]. Evidence from animal studies further revealed that feeding rats with arabinoxylan rice bran suppressed hepatocarcinogenesis by inhibiting cancer cell proliferation, suppressing inflammation, and inducing apoptosis [75]. A double-blind randomized control trial found that arabinoxylan rice bran enhanced the quality of life among neck and head cancer patients with chemoradiotherapy [76].

The combination of chemotherapeutic drugs with MGN/BioBran is likely to improve anticancer efficacy without increasing toxicity. The animal model study has demonstrated that combined paclitaxel (Taxol) and MGN-3/Biobran (40 mg/kg body weight) reduced the tumor volume and tumor growth in animals bearing Ehrlich ascites carcinoma cells [107]. The combination of paclitaxel (Taxol) and MGN-3/Biobran suppressed tumor proliferation by inducing apoptosis, increasing DNA damage, and increasing the sub-G_0_/G_1_ population [107]. This finding implies that MGN-3/BioBran sensitizes Ehrlich ascites carcinoma cells to paclitaxel by modulating the apoptotic pathway [107]. Various biological activities of MGN-3/Biobran have long been evaluated and have demonstrated its potential as a safe and non-toxic, anticancer agent [75].

In the context of colorectal cancer, rice bran oil has provided significant health-beneficial outcomes. Sirithunyalug et al. [77] evaluated the anti-inflammatory activity of natural purple rice bran oil derived from native Thai purple rice, including Khao’ Gam Boung, Khao’ Gam Thor, Khao’ Gam Pah E-Kaw, Khao’ Niaw Dam, and Khao’ Gam Leum-Phua in colorectal cancer cells. Natural purple rice bran oil was shown to be effective in the suppression of lipopolysaccharide (LPS)/interferon-γ (IFN-γ)-mediated induction of COX-2, iNOS, and nitric oxide [77]. Among the natural purple rice bran oil from the rice bran of Thai purple rice cultivars, natural purple rice bran oil derived from Khao’ Gam Leum-Phua showed the highest suppressive activity on nitric oxide and iNOS in RAW 264.7 cells [77]. The study further revealed that natural purple rice bran oil derived from Khao’ Gam Leum-Phua showed the highest suppressive activity on COX-2 expression in colorectal cancer cell lines including HCT-116 carcinoma cells and HT-29 adenocarcinoma cells [77]. The suppressive effect could be attributed to the unique complex of bioactive constituents such as tocotrienols, tocopherols, and γ-oryzanol [77]. Furthermore, the ethanolic extract of defatted rice bran also reduced the viability of breast cancer (MCF-7) and lung cancer (A549) cells [78]. The beneficial effects of rice bran and rice bran by-products could be partly modulated through additive/synergistic effects of bioactive compounds. Indeed, the bioactive constituents in rice bran demonstrated a better suppressive effect in cancer perhaps better than drugs, suggesting that whole food or whole food extract is crucial in the management of cancer [108,109]. Despite the clinical trial and preclinical findings have demonstrated a promising effect of rice bran in inhibiting cancer, the potential implications are worthy of further elucidation in long-term clinical trials.

### 5.3. Neurodegenerative Disease

Alzheimer’s disease is the most common form of neurodegenerative disease that is characterized by the progressive loss of cognition and memory. Alzheimer’s disease is the most common form of dementia and may contribute to 60–70% of cases [110]. Dementia affects language, calculation, learning capacity, judgment, comprehension, thinking, and orientation [110]. The deterioration in cognitive function is usually accompanied and occasionally preceded by impairment in motivation, social behavior, and emotional control [110]. Nearly 50 million people have dementia worldwide, of which about 60% live in middle- and low-income countries. Approximately 10 million new cases are reported annually [110]. The total number of individuals with dementia is expected to reach 152 million in 2050 and 82 million in 2030 [110].

Alzheimer’s disease is characterized by the presence of neurofibrillary tangles and senile plaques in the cortex and hippocampus of afflicted patients [111]. During the progression of Alzheimer’s disease, the brain tissues of these patients are exposed to oxidative stress. Advanced glycation end products exist in amyloid plaques and increased oxidation of glycated proteins may lead to extracellular accumulation [111]. Stimulation of the PPAR-γ has been reported to improve cognitive dysfunction and ameliorate inflammatory response in neurodegenerative disease [112]. Administration with PPAR-γ agonist improved cognitive performance, reduced Aβ production, and decreased neuroinflammation [112].

An emerging role for rice bran in response to neuroinflammation induced by oxidative stress has been demonstrated [68]. The implication of rice bran toward the pathophysiology of dementia and Alzheimer’s disease has been widely studied in in vivo models [80]. An animal study found that administration of 2% and 5% of rice bran for 13 weeks improved histological abnormalities and motor function, reduced oxidative stress, and increased α-tocopherol in the brain in vitamin E-deficient mice (Table 3) [79]. Intriguingly, vitamin E deficiency induced motor dysfunction caused by the degeneration of Purkinje cells in the cerebellum [79]. A recent study found that feeding Swiss albino mice with rice bran extract (100 mg/kg for 21 days) protects against the LPS-induced neuroinflammatory mouse model by reducing the inflammatory mediator levels in mice brains via targeting PPAR-γ nuclear receptor [80]. Another study performed by Saad El-Din et al. [81] also found that rice bran extract can mediate microglial phenotype from M1 to M2 and reduce the proinflammatory microglial marker (CD45) and NF-κB expression concomitantly with elevating the anti-inflammatory microglial levels and phagocytic markers (CD36, CD163, and arginase1). The study further revealed that rice bran extract significantly decreased p-tau protein expression and Aβ42 deposition and increased PPAR-γ levels [81], suggesting the possible role of PPAR-γ agonistic activity of rice bran extract in the treatment of neuroinflammation related to Alzheimer’s disease. In line with the study reported by Saad El-Din et al. [81], Mostafa et al. [82] also demonstrated that rice bran extract improved cognitive performance via modulation of PPAR-γ receptors in the neuroinflammatory mouse model.

In addition to the effects stated above, a beneficial effect of BioBran/MGN-3 has also been demonstrated on Alzheimer’s disease. Feeding streptozotocin-induced mice with BioBran/MGN-3 for 21 days upregulated antioxidant response element (ARE) and nuclear factor erythroid 2-related factor 2 (Nrf2), reduced intercellular adhesion molecule-1 (ICAM-1), IL-6, and malondialdehyde, and enhanced glutathione levels as well as reversed the spatial memory deficit [83]. The data further demonstrated that BioBran/MGN-3 possesses a protective effect toward amyloid-β induced apoptosis by upregulating the antiapoptotic Bcl-2 protein expression and suppressing cleaved caspase-3 and proapoptotic protein Bax, suggesting that BioBran/MGN-3 may protect against sporadic Alzheimer’s disease via stimulation of Nrf2/ARE and thereby mediates the amyloidogenic and apoptotic pathways [83]. Evidence from animal studies has demonstrated that rice bran oil (0.1, 0.5, and 1 mL/kg for 8 days) protects against Aβ (25–35)-induced memory impairment [84]. Amyloid β (25–35) leads to an increase in cholinesterase activity and lipid peroxidation and reduced glutathione in brain tissue [84]. Overall, rice bran and its derived components may alleviate oxidative stress and neurological disorders. The potential implication of rice bran on neurodegenerative diseases may be modulated partly through the additive/synergistic effect of bioactive constituents.

### 5.4. Cardiovascular Disease

Atherosclerosis, a common disease of the hardening of arteries, is one of the prevalent forms of CVD. Shear stress, hyperglycemia, dyslipidemia, chronic inflammation, and oxidative stress have been implicated in the pathogenesis of atherosclerosis [113]. High composition of phospholipids, cholesteryl esters, and cholesterol in low-density lipoprotein (LDL) are susceptible to oxidation modulated by reactive oxygen species (ROS). Indeed, oxidized LDL serves as a pro-atherogenic and pro-inflammatory modulator that triggers an inflammatory response and initiates endothelial dysfunction through inflammatory cytokines secretion and monocyte-derived macrophage-mediated synthesis [114]. Subsequently, this phenomenon stimulates recruitment and hence triggers the activation of macrophages as well as accumulates intracellular lipids within atherosclerotic lesions.

A study by Tan et al. [85] evaluated rice bran extract in relation to proinflammatory cytokine and inflammatory markers production. The study revealed that rice bran extract downregulated interleukin-6 (IL-6), inducible nitric oxide synthase (iNOS), tumor necrosis factor-α (TNF-α), interleukin-1β (IL-1β), and IL-1α, and ameliorates nitric oxide (NO) overproduction in LPS-treated murine J774A.1 macrophage-like cells (Table 3) [85]. The animal study further demonstrated that rice bran extract significantly decreased pro-atherogenic oxidized LDL/β2-glycoprotein I (oxLDL/β2GPI) complexes, triglycerides (TG), and total cholesterol (TC) plasma levels in high-fat diet-induced low-density lipoprotein receptor knockout (*Ldlr*^−/−^) mice, implied that the rice bran extract attenuates the risk of oxidative stress and chronic inflammation underlying the pathogenesis of atherosclerosis [85]. In support of this, rice bran extract also modulates proinflammatory mediator overproduction by inducing activation of ARE and mitogen-activated protein kinase (MAPK) and suppressing NF-κB signaling pathways [85]. This favorable effect could be attributed to the synergistic protective effect of vitamins, polyphenols, and γ-oryzanol in rice bran extract.

Emerging studies have demonstrated that tocotrienols, oryzanols, and phytosterols exert specific hypocholesterolemia activity. Gamma-oryzanol was found to be inversely linked to cholesterol serum and plasma levels as well as reduced cholesterol absorption, and subsequently lower hyperlipidemia [115]. A randomized double-blind controlled trial involving 59 hyperlipidemic participants found that the intake of a diet with 30 mL/day of rice bran oil (containing 11,000, 8000, or 4000 ppm γ-oryzanol) for 4 weeks significantly reduced LDL-C levels and improved antioxidant status compared to the control, suggesting that rice bran oil may alleviate CVD risk factors [86]. This favorable effect could be due to the activity of rice bran oil which is involved in the suppression of the crucial regulatory enzymes of the cholesterol biosynthesis pathway including 3-hydroxy-3-methylglutaryl coenzyme A (HMG-CoA) reductase. Gamma-oryzanol may reduce the blood lipids and decrease the absorption of cholesterol by suppressing the activity of cholesterol 7-alpha-hydroxylase [86]. Furthermore, data from a meta-analysis and systematic review of randomized controlled trials involving a study from inception to 7 October 2020 further demonstrated that rice bran oil consumption reduced triglycerides, LDL-C, and total cholesterol serum levels, suggesting that rice bran oil may decrease the hyperlipidemia/dyslipidemia risk [116].

### 5.5. Diabetes

Nearly 422 million people have diabetes globally, in which the majority are living in middle- and low-income countries [117]. About 1.6 million deaths are attributed to diabetes every year [117]. The prevalence of diabetes mellitus is expected to increase up to 642 million by the year 2040 [118]. Diabetes mellitus is a multifaceted metabolic disorder characterized by high blood glucose levels (hyperglycemia), which is caused by insulin resistance or insufficient insulin production. The damage in the β-cells of the pancreatic islets decreases insulin secretion and subsequently leads to resistance to the action of insulin [119]. It can also lead to serious complications such as diabetic retinopathy, neuropathy, and nephropathy.

Compared to phenolics-removed rice bran dietary fiber, rice bran dietary fiber and formulated rice bran dietary fiber (obtained by mixing hydrolyzed-bound phenolics and phenolics-removed rice bran dietary fiber) significantly decreased fasting blood glucose levels (Table 3) [87]. The presence of bound phenolics could stimulate the GLUT4/Akt/IRS1 insulin signaling pathway in skeletal muscle and modify gut microbiota by mediating gut microbiota dysbiosis and enriching the butyric acid-producing bacteria genera of the families *Ruminococcaceae* and *Lachnospiraceae*, and thereby resulting in the decreased of blood glucose levels. Evidence from this study suggests that bound phenolics may contribute to the antihyperglycemic effect of rice bran dietary fiber [87].

Diabetic nephropathy is the most prominent cause of end-stage renal disease globally, in which type 2 diabetes patients with diabetic nephropathy contributed to 42% of cases [120]. Thus, it is crucial to prevent the progression of diabetic nephropathy and diabetes to end-stage renal disease. Dietary protein restriction is thought to be the most effective nutritional approach in delaying this progression [121], despite inconsistent research evidence. Although the effects of different types of dietary proteins on kidney disease have not been well studied, it has been demonstrated that plant proteins delay the progression of kidney disease [122]. An earlier study has shown that the intake of rice endosperm protein significantly inhibits renal tissue damage and urinary albumin excretion in Zucker diabetic fatty and Goto-Kakizaki rats [123]. A recent study by Kubota et al. [88] evaluated the effects of rice bran protein in relation to diabetic nephropathy and diabetes in Zucker diabetic fatty rats. The data showed that feeding Zucker diabetic fatty rats for 8 weeks with rice bran protein significantly improved the plasma adiponectin levels and hemoglobin A1c. The study further showed that histological damage, N-acetyl-β-_D_-glucosaminidase, and urinary albumin excretion in the kidney were significantly reduced in rice bran protein-fed rats. Evidence from this study suggests that a diet containing rice bran protein has beneficial effects on hepatic lipid accumulation, diabetic nephropathy, and diabetes [88]. The renoprotective effects of rice bran protein could be achieved secondary by improving blood glucose control. There may have been direct exposure to bioactive peptides derived from rice bran protein or specific amino acids. With regard to bioactive peptides, rice bran protein hydrolysate prepared with protease G6 improves the urinary albumin/creatinine ratio, decreases fasting glucose levels, and improves insulin sensitivity in diabetic mice [124]. Nonetheless, the favorable effect on diabetic nephropathy may not be due to the specific peptides; the hydrolysate may compose of a mixture of amino acids, for instance, arginine, and peptides [124].

Glucose homeostasis is mediated by a series of events within the pancreatic β-cells and leads to insulin secretion [125]. Nonetheless, impairment of glucose-stimulated insulin action due to inflammation and oxidative stress can lead to insulin resistance and β-cell dysfunction, subsequently leading to the pathogenesis of type 2 diabetes mellitus [126]. Rice bran extracts (25–250 μg/mL) significantly stimulated the glucose-stimulated insulin secretion and *Ins1*, *Tfam*, *Sirt1*, *Pdx1*, and *Glut2* gene expression under high glucose and normal conditions [127]. Rice bran phenolic extracts favorably mediated the transcriptional activity involved in insulin secretion and β-cell dysfunction via a few mechanisms such as activation of survival and effectors factors of insulin secretion, decreasing free radical damage by its antioxidant activity, and synergistic action of polyphenols targeting signaling molecules [127], suggesting that rice bran phenolic extracts have a great promise in the treatment of glucotoxicity induced β-cell dysfunction. Emerging evidence has indicated that pigments in rice bran layers have beneficial biochemical and antioxidant effects against oxidative stress-induced diseases including diabetes (Seechamnanturakit et al., 2018). Pigmented rice has naturally occurring colored components that belong to the flavonoid group, namely anthocyanins [128]. An animal study by Nakamura et al. [89] demonstrated that super-hard rice bread blended with black rice bran suppressed the abrupt increase in postprandial blood glucose levels. The proteomic profiling analysis also indicates that purple rice bran could increase insulin sensitivity and promote hepatic glucose uptake as well as suppress hepatic gluconeogenesis [129].

## 6. Application in Food Products

Rice bran contains various bioactive substances and nutrients including vitamins, lipids, protein, γ-oryzanol, phenolic compounds, and trace minerals [7]. Evidence from epidemiological studies suggests that oxidative stress and chronic diseases can be mediated by nutrient-rich antioxidants [130]. Due to its functional and nutritional properties, a broad spectrum of food applications in rice bran has been identified (Table 4). Over the past decades, consumers are concerned about well-being and fitness and prefer to consume nutritious food. Subsequently, food manufacturers are looking forward to producing functional foods that could improve individual health and prevent metabolic ailments. Several studies reported by Tan and Norhaizan [10] indicate that rice bran by-products and rice bran have unique properties, making them suitable for use in the food, pharmaceutical, and nutraceutical industries. Rice bran in the form of protein concentrates, full-fat rice bran, rice bran oil, and defatted rice bran exerts bioactive constituents that could be functional ingredients for food production (Figure 3).

### 6.1. Cooking Oils

Rice bran oil, an oil extracted from the bran layer of the rice kernel, has a pleasant smell and mild flavor that can be utilized as an essential food ingredient in various cuisines worldwide [149]. A study reported by Lai et al. [150] found that rice bran oil exerts a high ignition point (~350 °C) and smoke point (232 °C), suggesting that rice bran oil is stable with a low level of polymerization and degradation during cooking that may fit for high-temperature cooking methods. The production of rice bran oil is sustainable with a hypoallergenic profile and thus can be utilized to substitute traditional cooking oil for those who are allergic [151]. Compared to vegetable oils, rice bran oil shows a slow increase in total polar compounds, degree of polymerization, and peroxide value upon deep-frying [151]. Another unique feature of rice bran oil is its viscosity. Diamante and Lan [152] evaluated the viscosities of vegetable oils including walnut oil (cold pressed), sunflower oil, soybean oil, sesame oil, safflower oil (cold pressed), rice bran oil, rapeseed oil (cold pressed), peanut oil, olive oil (mixture of refined and cold pressed), macadamia nut oil (cold pressed), grape seed oil, canola oil, and avocado oil (cold pressed). The data showed that rice bran oil is the most viscous compared to other oils studied [152], implying that rice bran oil has an excellence dressing performance, particularly in Chinese cuisine. Rice bran oil can be easily retained on food surfaces due to its high viscosity, thus leading to appetizing and shiny food [149].

### 6.2. Bakery Products

The addition of full-fat rice bran flour into the biscuits and bread significantly improved the mineral contents (zinc, iron, calcium, sodium, and potassium) and proximate composition (ash, crude fiber, crude fat, and crude protein) compared to the wheat flour [146]. However, the data revealed that adding full-fat rice bran flour from 0 to 20% slightly decreased the specific loaf volume of bread [146]. The reduction in the specific loaf volume of bread could be attributed to the decrease in wheat protein (gluten), and subsequently decreased air entrapment in the dough [153]. Sensory analysis showed that 10% of full-fat rice bran flour can be incorporated into the bread and 20% into biscuit productions with acceptable sensory and physicochemical attributes [146]. In line with this, the study also found that incorporating bran in bread reduced the bread volume and elasticity of crumbs [154]. Research evidence indicates that sourdough fermentation could improve bread’s shelf-life, and structural and nutritional properties [155]. The substitution of wheat flour with 10% rice bran sourdough increased the bread volume [145]. Incorporating rice bran sourdough into wheat flour improved the crumb texture of bread [145]. Rice bran sourdough bread was significantly softer compared to the bread loaves produced from 100% wheat flour (control) [145]. This favorable effect could be due to the sourdough fermentation which plays a crucial role in preserving bread freshness by affecting the load moisture redistribution during storage via acidification [156]. Rice bran sourdough bread was shown to be the best sensory in terms of texture, taste, appearance, overall acceptability, and color compared to wheat bran sourdough bread, wheat bran bread, and rice bran bread [145]. This finding implies that *Lactobacillus plantarum* fermented rice bran sourdough could be used as an alternative approach for sourdough bread production [145]. During the fermentation of sourdough, the activation of proteolysis and acidification occurs. Subsequently, this process causes biochemical changes and affects the baked products and dough matrix, thus improving the nutritional quality and function of baked products [157]. Rice bran fermented with yeast also showed a better nutritional value such as protein compared to non-fermented rice bran [158].

The incorporation of rice bran into wheat flour not only improved the protein contents but also enhanced the phenolic compounds in cookies. The cookies containing 100, 50, and 25 g of fermented rice bran demonstrated higher antioxidant activity, phenolic compounds, and protein levels compared to the control cookies [143]. The sensory evaluation demonstrated that incorporating fermented rice bran into the cookies presented good acceptability and sensory attribute, with a preference for the cookies containing 25 g of fermented rice bran [143]. This result implies that rice bran fermented by *Saccharomyces cerevisiae* could be utilized as an alternative ingredient to prepare gluten-free cookies [143]. A study by da Rocha Lemos Mendes et al. [132] evaluated the effects of replacement wheat flour with different amounts of defatted rice bran on sensory, technological, nutritional, and physicochemical composition profiles in cakes. The sensory analysis showed that the substitution of wheat flour with 30% of defatted rice bran exhibited higher acceptance levels. This study further demonstrated that the incorporation of defatted rice bran into the cakes increased antioxidant capacity, phenolic compounds, and fiber content, and reduced the energy value [132]. This finding suggests that defatted rice bran could be used as a raw material to improve the nutritional values of bakery products.

### 6.3. Food Colorant

Food color is a visual element that arises from the spectral distribution of light, which is an important indicator of food quality [159]. Food color is one of the predominant factors that directly attract consumers’ interest, improve the appearance of food products, and eating desires [160]. The color additives are used to restore the color of food lost during the storage and processing of food. Colorants are often used in processed meat products, snacks, beverages, sauces, and margarine [161]. Color additives are any substance, pigment, and dye that are capable of coloring [162]. However, different views emerged when food additives are evaluated in terms of health risks. The incidence of asthma and allergies is increased in recent decades, and these incidences are linked to food additives, and particular colorants [163]. It has been shown that synthetic food additives increased asthma and urticaria in some individuals [164]. Indeed, most consumers are concerned about naturalness as a vital property. Therefore, natural foods are considered to be healthier and safer than artificial food [165].

Although white rice is consumed as a predominant staple food worldwide, some countries in Southeast Asia also consume pigmented cultivars, such as red, black, purple, and brown rice. The color intensity of pigmented rice is obtained from the value of yellowness, redness, and lightness may link to the bioactive constituents. Proanthocyanidins and anthocyanins belong to plant flavonoids, which are believed to be the predominant functional components present in purple, red, and black rice. The colorant powder obtained from the black glutinous rice bran using ohmic heating-assisted extraction had a higher bioactive constituent, anthocyanin pigment, and colorant yield compared to conventional procedures [166]. Anthocyanins not only exert natural color but also provide a broad spectrum of health-promoting effects on humans and animals [167]. Feeding aged mice with a diet containing super-hard rice bread blended with black rice bran for 4 weeks decreased amyloid-β 40 peptides compared to the mice fed with a commercial diet, suggesting that super-hard rice bread blended with black rice may inhibit amyloid β production [89]. Furthermore, Nakamura et al. [89] also found that super-hard rice bread blended with black rice bran contained high levels of resistant starch and demonstrated strong suppressive activity against acetylcholinesterase and β-secretase even after heating.

Research evidence has revealed that red and black rice bran contains high antioxidant activities and phenolic compounds [168]. Loypimai et al. [134] studied the natural colorant of black rice bran on antioxidant activity, bioactive compounds, lipid oxidation, and color characteristics of fermented Thai pork sausage. Adding black rice bran colorant powder to the fermented sausages increased bioactive constituents and improved color formation [134]. Compared to the sausages containing 120 ppm of nitrite, sausages with black rice bran colorant powder showed a higher level of antioxidant, total phenolic, and anthocyanin [134]. In general, nitrite/nitrate is often used to inhibit the proliferation of pathogenic bacteria such as *Listeria* spp. and *Clostridium botulinum*. Nitrate/nitrite can form nitrosamine when interacting with a secondary amine in the stomach, which is carcinogenic to humans. The data suggest that black rice bran colorant powder is an excellent source of anthocyanin pigments and may partially replace nitrite in fermented sausage products [134].

### 6.4. Edible Coating

The edible coating is a thin layer of edible substance applied to the surface of food products to provide barriers to solute movement, oxygen, and moisture. The edible coating is widely utilized in fresh vegetables and fruits to maximize the shelf-life and quality by decreasing solute and moisture migration, oxidative reaction rates, respiration, and gas exchange. A study showed that coating rice bran wax in bread significantly reduced crumb hardening and moisture loss [140].

Rice bran wax can be used as an alternative coating or protective layer for fruits and vegetables. Rice bran wax has good applicability in the formation of lipid-based edible coatings, after the removal and refining of crude resinous substances [139]. It is a hydrophobic matter, and thus it can decrease moisture loss from food products during storage. The use of rice bran wax as a coating substrate can be utilized for the shelf-life extension of horticultural crops and delay the ripening process by mediating the immediate environment of the food products [139]. A study demonstrated that adding 10% rice bran wax emulsion to tomatoes showed a better shelf-life (27 days) than the control sample (18 days) [139]. Zhang et al. [135] also found that the weight loss of rice bran wax-coated cherry tomatoes was lower (13.54%) compared to the control (16.02%). The study further demonstrated that rice bran wax coating prevented the degradation of chelate-soluble pectin [135]. Evidence from this study suggests that rice bran wax coating could serve as an effective approach to preserving fresh fruits [135].

In a further study focused on defatted rice bran, a by-product produced from the rice bran oil industry, Chayawat and Rumpagaporn [131] compared four defatted rice bran substitution levels (20%, 15%, 10%, and 0% of mixed flour in batter and pre-dust) on the properties of the fried chicken nugget. The data demonstrated that higher levels of defatted rice bran substitutions increased batter viscosity, thus leading to a thicker nugget crust. Moreover, the oil content is significantly reduced while fiber and moisture content increased in defatted rice bran batter-coated chicken nuggets compared to those without defatted rice bran, suggesting that 15% of less defatted rice bran substitution in batter decreases fried chicken nugget oil content while maintaining healthfulness and product quality [131].

In addition to rice bran wax and defatted rice bran, rice bran dietary fiber has the potential to be used to improve food product quality. Jiang et al. [169] compared Asian pear powder encapsulated with maltodextrin and rice bran dietary fiber, respectively. The study has demonstrated that the addition of rice bran dietary fiber in Asian pear powder showed higher antioxidant activity and total and bioaccessible phenolics compared to that of Asian pear powder encapsulated with maltodextrin [169]. Intriguingly, high retention of bioactive constituents was reported in Asian pear powder encapsulated with rice bran dietary fiber during storage for 150 days [169].

## 7. Conclusions and Future Perspective

This review has shown clear evidence that intake of rice bran and its derived compounds may offer optimal health in in vitro and in vivo models as well as human studies. Therefore, rice bran holds great promise and may provide a useful approach to improving the immune system, alleviating chronic diseases, scavenging ROS, and decreasing inflammation. The broad spectrum of processes in which the antioxidants and bioactive compounds are involved suggests the protective role of rice bran and its derived compounds in the pathogenesis of several metabolic ailments. Indeed, rice bran exerts many bioactive constituents, for instance, phenolics, γ-aminobutyric acid (GABA), squalene, phytosterols, tocopherols, tocotrienols, and γ-oryzanol. Due to the therapeutic potential, incorporating rice bran in food can contribute to the development of value-added foods or functional foods that currently are in high demand. Indeed, the addition of rice bran and its derived compounds has been utilized as supplements in many food matrices as well as improves the nutritional quality of processed food. Therefore, rice bran produced from the rice milling process or rice bran oil production may improve the economy of the rice-producing nations. However, there are several limitations in this study including (1) small sample size; (2) short duration of treatment; (3) different daily dosages of rice bran; and other factors such as the age of animals and subjects of the study. In addition, assessment of dose–response relationship and chronic diseases in clinical trials has not been evaluated. Collectively, this review may pave the way for the potential use of rice bran as a functional ingredient in various foods and to combat chronic diseases. The crucial role played by rice bran is nonetheless worth studying in-depth in long-term clinical trials.

## Figures and Tables

**Table 1:** The projection of population in rice-consuming and producing countries in Asia from 1995 to 2025.

Country	Population (Million) 1995	Annual Growth Rate (%)		Percentage Increase 1995–2025	Projected Population (Million) in 2025
		2020–2025	1995–2000		
Asia (excluding China)	2244	1.1	1.8	51	3389
China	1199	0.5	0.9	23	1471
India	934	1.0	1.7	47	1370
Indonesia	192	0.8	1.4	38	265
Pakistan	130	1.6	2.7	87	243
Japan	125	−0.3	0.3	−1	124
Bangladesh	121	1.1	1.8	50	182
Vietnam	74.1	1.2	2.0	58	117
Philippines	69.2	1.2	2.2	66	115
Thailand	60.5	0.7	1.3	34	80.8
Myanmar	46.8	1.1	2.1	56	72.9
Republic of Korea	44.8	0.3	0.8	18	52.9

Source: FAO [18].

**Table 2:** The effect of phytochemicals on metabolic ailments.

Phytochemical	Metabolic Ailments	Findings	References
Tocopherols and tocotrienols	Cardiovascular disease	No remarkable effect on the lipid profile in menopausal women	[49]
		Did not affect blood lipid parameters in patients with diabetes	[50]
		Reduce lipoprotein-lipid profile	[51]
	Neurodegenerative disease	Protect mouse hippocampal neurons from apoptosis	[52]
Gamma-oryzanol	Cardiovascular disease	Decreased secretion of IL-1β by peritoneal macrophages	[53]
		Increased expression of IL-10 and IL-4 compared to control group	[54]
	Cancer	Decreased inflammatory response by reducing NF-κB transcriptional activity	[55]
γ-aminobutyric acid	Type 2 diabetes mellitus	Improved hyperglycemia	[56]
	Cardiovascular disease	Decreased risk of cardiovascular disease	[57]
	Hypertension	Only observed during acute administration but no effect after chronic administration	[58]
Polyphenols	Cardiovascular disease	Prevent cardiovascular disease	[59]
		Exert pro-oxidant effect in Cu^2+^-induced oxidation of LDL	[60]
	Type 2 diabetes mellitus	Improved insulin action and β-cell function	[61]
		Decreased α-glycosidase, α-amylase, and AR enzymes	[62]

AR: aldose reductase; IL-1β: interleukin-1beta; IL-4: interleukin-4; IL-10: interleukin-10; LDL: low-density lipoprotein; NF-κB: nuclear factor-kappa B.

**Table 3:** The effects of rice bran and their derived compounds on metabolic ailments.

Metabolic Ailments	Cell Lines/Animal Studies/Subjects	Treatment	Findings	References
Hypertension	Stroke-prone spontaneously hypertensive rats	Fermented rice bran	Improved high blood pressure after 6 h of administration	[69]
	Individuals with grade 1 hypertension or high-normal blood pressure	Rice bran supplement containing processed rice bran	Reduced in systolic blood pressure	[70]
	Spontaneously hypertensive rats	Rice bran protein hydrolysates 1A <3 kDa	Decreased systolic blood pressure	[71]
	Spontaneously hypertensive rats	Leu-Arg-Ala, from thermolysin-digested rice bran	Decreased SBP levels	[72]
Cancer	Prostate cancer PC-3 cells	Cyanidin 3-glucoside derived from rice bran	Reduced the percentage of cell survival and decreased the expression of p-FAK and β-catenin	[73]
	H1299 non-small-cell lung cancer-bearing mice	Defatted rice bran and rice bran polysaccharides	Inhibited H1299 non-small-cell lung cancer	[74]
	Rats	Arabinoxylan rice bran	Inhibited cancer cell proliferation, suppressed inflammation, and induced apoptosis	[75]
	Head and neck carcinoma patients undergoing radiation therapy	Arabinoxylan rice bran	Enhanced quality of life	[76]
	Colorectal cancer cells including HCT-116 carcinoma cells and HT-29 adenocarcinoma cells	Natural purple rice bran oil	Suppressed LPS/IFN-γ-mediated induction of COX-2, iNOS, and nitric oxide	[77]
	Breast cancer (MCF-7) and lung cancer (A549) cells	Ethanolic extract of defatted rice bran	Reduced the viability of MCF-7 and A549 cells	[78]
Neurodegenerative disease	Vitamin E-deficient mice	2 and 5% of rice bran	Improved histological abnormalities and motor function, reduced oxidative stress, and increased α-tocopherol in the brain	[79]
	Swiss albino mice	Rice bran extract	Reduced inflammatory mediator levels in mice brains	[80]
		Rice bran extract	Reduced proinflammatory microglial marker (CD45) and NF-κB expression	[81]
	Neuroinflammatory mouse model	Rice bran extract	Improved cognitive performance	[82]
	Streptozotocin-induced mice	BioBran/MGN-3	Upregulated ARE and Nrf2	[83]
	Mice	Rice bran oil	Protects against Aβ (25–35)-induced memory impairment	[84]
Cardiovascular disease	Murine J774A.1 macrophage-like cells	Rice bran extract	Downregulates IL-6, iNOS, TNF-α, IL-1β, and IL-1α, and ameliorates NO overproduction	[85]
	High-fat diet-induced low-density lipoprotein receptor knockout (*Ldlr*^−/−^) mice	Rice bran extract	Significantly decreased pro-atherogenic oxLDL/β2GPI complexes, TG, and TC plasma levels	[85]
	Hyperlipidemic participants	Rice bran oil	Reduced LDL-C levels and improved antioxidant status	[86]
Diabetes	db/db mice	Rice bran dietary fiber	Significantly decreased fasting blood glucose levels	[87]
	Zucker diabetic fatty rats	Rice bran protein	Significantly improved plasma adiponectin levels and hemoglobin A1c	[88]
	Mice	Super-hard rice bread blended with black rice bran	Suppressed the abrupt increase in postprandial blood glucose levels	[89]

ACE: angiotensin-converting enzyme; ARE: antioxidant response element; COX-2: cyclooxygenase-2; IFN-γ: interferon-γ; IL-1β: interleukin-1β; IL-6: interleukin-6; iNOS: inducible nitric oxide synthase; LDL-C: low-density lipoprotein cholesterol; LPS: lipopolysaccharide; NF-κB: nuclear factor kappa B; NO: nitric oxide; Nrf2: nuclear factor erythroid 2-related factor 2; oxLDL/β2GPI: oxidized LDL/β2-glycoprotein I; SBP: systolic blood pressure; TC: total cholesterol; TG: triglycerides; TNF-α: tumor necrosis factor-α.

**Table 4:** The potential application of rice bran in the food industry.

Rice Bran	Purpose of Addition	Food Products	References
Defatted rice bran	Reduced oil content	Chicken nugget	[131]
Defatted rice bran	Increased antioxidant capacity, phenolic compounds, and fiber content	Cakes	[132]
Bleached rice bran wax	Improved hardness and color of cookies	Cookies	[133]
Black rice bran colorant powder	Increased bioactive constituents and improved color formation	Fermented Thai pork sausage	[134]
Rice bran wax	Decreased weight loss	Cherry tomatoes	[135]
Rice bran wax	Inhibit lipid oxidation	Frankfurter-type sausages	[136]
Rice bran wax	Improved oil-binding capacity and increased firmness	Peanut butter	[137]
Rice bran wax	Enhanced stability	Peanut butter	[138]
Rice bran wax	Extend shelf-life	Tomatoes	[139]
Rice bran wax	Reduced crumb hardening and moisture loss	Bread	[140]
Rice bran oil	Enhanced stability	Margarine	[141]
Rice bran oil	Improved oxidative stability	Fat spreads	[142]
Fermented rice bran	Increased antioxidant activity, phenolic compounds, and protein levels	Cookies	[143]
Rice bran insoluble dietary fiber	Reduced in vitro starch digestibility	Noodles	[144]
Rice bran	Increased bread volume	Sourdough bread	[145]
Full fat rice bran	Improved mineral contents (zinc, iron, calcium, sodium, and potassium) and proximate composition (ash, crude fiber, crude fat, and crude protein)	Bread and biscuits	[146]
Rice bran	Increased gumminess and firmness	Bread	[147]
Rice bran fiber	Enhanced total dietary fiber, insoluble dietary fiber, and soluble dietary fiber contents	Rice pasta	[148]
